# Latino gangs in Spain: from the Latin Kings & Queens to the Dominican don’t play

**DOI:** 10.1080/13691457.2024.2352001

**Published:** 2024-05-16

**Authors:** Carles Feixa

**Affiliations:** Department of Communication, Pompeu Fabra University, Barcelona, Spain

**Keywords:** Gangs, Latin Kings & Queens, Dominican don’t play, moral juvenicide, mediation, Pandillas, Latin Kings & Queens, Dominican Don’t Play, Juvenicidio moral, Mediación

## Abstract

In October 2003, a young Colombian was murdered after he left the high school where he was studying in Barcelona after an alleged confrontation between two gangs composed of young people of Latin American origin – the Ñetas and the Latin Kings & Queens. This led to a wave of moral panic over the presence in Spain of the so-called ‘*bandas latinas*’ (Latino gangs). Eighteen years later, in February 2021, two further murders in Madrid linked to clashes between two other gangs – Dominican Don't Play and Trinitarios – generated alarm and led to a campaign with important media, political, legal, and criminal repercussions. This text offers a review of two decades of the presence of street youth organisations of Latin American origin in Spain, presenting the first results of the TRANSGANG project, focusing on experiences of mediation with these groups, and ends with a discussion on ‘Latino gangs’ as a symptom of ‘moral juvenicide.’

## RESUMEN

En octubre de 2003, un joven colombiano fue asesinado tras salir del instituto donde estudiaba en Barcelona tras un presunto enfrentamiento entre dos pandillas compuestas por jóvenes de origen latinoamericano: los Ñetas y los Latin Kings & Queens. Ello provocó una oleada de pánico moral por la presencia en España de las llamadas “bandas latinas”. Dieciocho años después, en febrero de 2021, otros dos asesinatos en Madrid vinculados a enfrentamientos entre otras dos pandillas –Dominican Don’t Play y Trinitarios– generaron alarma y desembocaron en una campaña con importantes repercusiones mediáticas, políticas, jurídicas y penales. Este texto ofrece un repaso a dos décadas de presencia de organizaciones juveniles callejeras de origen latinoamericano en España, presentando los primeros resultados del proyecto TRANSGANG, centrándose en experiencias de mediación con estos colectivos, y finaliza con una discusión sobre las “bandas latinas” como síntoma de “juvenicidio moral”.

## Introduction: from ‘Youth Gangs’ to ‘Latino Gangs’

Anyone who has studied gangs over a period of time will admit that the more they are studied, the more complex they are. At best, we can come to understand a little about certain characteristics of gangs at certain times. Gangs are dynamic, flexible and constantly changing. (Sanders, 1994, p. XI).

On 28 October 2003, Ronny Tapias, a young man of Colombian origin living in Barcelona, was murdered by being stabbed in a fight between rival groups at the exit of the high school where he was studying. The media immediately linked this to a confrontation between two ‘*bandas latinas*’ (Latino gangs) – the Latin Kings^1^ and the Ñetas^2^ – a term that has since become popular, generating a ‘moral panic’ that spread throughout Spain (and is somehow still present almost twenty years later). In the trial that took place at the Provincial Court of Barcelona in June 2005, whose sessions I followed live, the link between the fight and the gangs was not proven. Before the trial, the prosecutor called me to advise her after consulting my book *De jovenes, bandas y tribus* (On Youth, Gangs and Tribes) (Feixa, 1998), since in the statements associated with the testimonies, many other names of gangs appeared – Rancutas, Vatos Locos, Black Panthers, etc. – of which some of the accused – a dozen young people of Ecuadorian and Dominican origin – acknowledged being members. In the trial, it was said that they had confused Ronny with a Latin King with whom they had fought in a Latino nightclub (significantly called Caribe Caliente) the previous weekend. In 2012, I interviewed one of the young people who was serving a sentence for the murder in the prison of Can Brians. He confessed to me that he was neither a Latin King nor a Ñeta but a member of the Black Panthers, but that in reality, the fight had more to do with a conflict over a girl in the discotheque; that the aggrieved had convinced his colleagues to go with him to chastise the offender at the high school where he studied, and that without having planned it, someone took out a knife and tragedy happened.

Be that as it may, Ronny's death fueled the myth of ‘Latino gangs,’ which have since come to be considered a ‘public enemy’ by the media, public opinion, the police, and prosecutors (at the meeting, the chief prosecutor asked me if tattoos could be a clear indication of gang membership in an attempt to import the legislation in force in El Salvador, which allowed individuals to be arrested just for wearing tattoos, which fortunately did not happen). Paradoxically, the victim of the murder was alleged to be a member of the Latin Kings, but this gang was also blamed, uniting executioners and victims under the same umbrella. The high school where the tragedy happened, in a middle-class neighbourhood of Barcelona's city centre – until then prestigious – became a victim of stigma and soon after a school ghetto, like so many other high schools on the urban periphery that I visited during those years. The director contacted me from time to time to help him reverse the situation, but the Department of Education of the Catalan Government finally decided to close the centre, given the persistent reduction in enrollment (Feixa et al., 2008; Feixa & Andrade, 2020).

In the early hours of Saturday, February 5 to Sunday, 6 February 2022, two young people of Latin American origin, Jaime Guerrero and Diego Fernando López, were killed in Madrid. Jaime was fifteen years old. Diego was twenty-five years old and of Colombian origin, although he possessed Spanish nationality; according to press agencies, he had a history of making threats, theft, tumultuous brawls, and illegal possession of weapons. The same night, the police arrested two of Diego's alleged murderers, Sandy Antonio Campusano, a twenty-one-year-old Dominican boy, who had been arrested two months ago for a prostitution plot involving girls from juvenile centres, and a twenty-seven-year-old Ecuadorian boy, also with a criminal history, who was arrested bloodied. According to the police report, that same night, they had gone with a group of about twenty young people armed with knives and ‘*machetes*’ (big knives) to ambush another group – a ‘*caída*’ (fall) in the slang used by these groups –, who were drinking in the street. The confrontation was allegedly due to a tumultuous fight between Dominican Don’t Play (DDP)^3^ and Trinitarios (3Nis),^4^ two ‘Latino gangs’ of Dominican origin who had participated in a series of confrontations in Madrid that led to deaths.

The event generated a moral panic similar to or even greater than that aroused by the murder of Ronny Tapias, with excessive media attention: the day after the murder, several media representatives called me, and during the following weeks, we were constantly requested to offer our opinion to the press, radio, television and digital media. The weekend after the murder, the police set up an intervention with several hundred officers stationed in the neighbourhoods where the members of the two gangs allegedly lived, and more than a hundred arrests were made. The version of the story given to us by some of our informants, as well as the educators who work with them, was very different and alluded to feelings of harassment and marginalisation, as well as the role of rumors and hate speech fueled by extreme right-wing parties, which encouraged groups of minors who were not even from the gangs to meet to fight. Despite the extensive police interventions and the constant raids, a few months after the death of Jaime and Diego, there was another death directly related to the previous ones, supposedly in revenge for them. In fact, according to our data, the first death was related to another murder that had occurred near Puerta del Sol in 2016, which in turn was the result of previous murders that took place in the 2000s and consolidated the rivalry between the two gangs. However, instead of mediation and preventive measures, police persecution and harassment were the chosen approach, with results known to all.

Almost twenty years have passed between Ronny's death and those of Jaime and Diego. Although the social context and that of the gangs is very different, the social, police, and media reaction in both cases are associated with remarkable parallels. The two decades that separate the events mark the transition from Generation 1.5 to Generation 2.0. While the first primarily involved teenagers and young people who had recently arrived in Spain due to family reunification, the second was mainly composed of people born and raised there. However, cyberspace connects them transnationally (hence the dual meaning of 2.0). This article aims to reflect on the continuities and changes in youth gangs and the policies deployed to confront them, trying to reach beyond the stereotypes. To do this, I will begin by going back to the origin of the youth gangs in Spain in the 1960s and 1970s; I will then introduce the emergence of the so-called ‘*bandas latinas*’ (Latino gangs) from the year 2000, presenting the mediation process with these groups in Barcelona in which I had the opportunity to participate from 2005 to 2009. I will conclude by outlining the current situation, which constitutes the immediate context of the two murders mentioned above.

## Theoretical and methodological framework: researching transnational gangs

The present study is based on four decades of research on youth street groups – the so-called ‘gangs’ – in Spain and other countries, which began with an oral history of youth subcultures in Catalonia, and continued with a classic ethnography about a punk gang in Mexico City (Feixa, 1998). Then it focuses on the results of several projects about ‘Latino gangs’, initiated with a commission from the Barcelona City Council after the murder of Ronny Tapias through the JOVLAT project (Feixa et al., 2006), and culminated with the TRANSGANG project on mediation experiences with youth street groups in 12 cities in southern Europe, northern Africa and the Americas (Feixa et al., 2023). In all these projects, a participatory action research methodology has been applied, starting from fieldwork that includes participant observation and in-depth interviews with gang members and stakeholders, combined with the direct participation of the groups investigated, which implies the moving from ‘applied research’ to ‘involved research’. In the case of the JOVLAT project, this translated into my direct participation as a ‘facilitator’ in the constitution of youth associations, a process described below. In the case of the TRANSGANG project, it consisted of carrying out mediation courses and producing documentary films and music videos with the members of the groups. In all cases, the common thread was the collection of life stories co-authored with the protagonists, some of which have been published (Feixa & Andrade, 2020; Feixa & Valle, 2022).

From a theoretical point of view, the concepts that frame the article are those of transgang, mediation and moral juvenicide. The notion of *transgang* refers to the globalisation of gang culture, as a consequence of migratory processes, ‘iron fist’ policies, and the transfer from the street corner to virtual space (Feixa et al., 2019). The notion of *mediation* refers to the experiences of conflict resolution within groups, between groups and with the social environment, using strategies such as ‘care mediation’ (promoted by the ‘left hand’ of the State) and ‘mutuality mediation’ (promoted by gang leaders and NGOs), and tactics such as rap battles, the use of video or the constitution of associations (Feixa et al., 2023). The notion of *moral juvenicide*, finally, is based on the concept of necropolitics (Mmembe, 2003) and refers to the processes of stigmatisation, symbolic suppression, and discrimination based on origin, race, age, and gang affiliation, which translates into feelings of precarity, fatalism and hopelessness (Valenzuela, 2015). In the discussion section we will return to the intersectional dimensions of this triad.

## Antecedents of youth gangs in Spain: from the ‘*golfos*’ to the ‘*quinquis*’

The main European capitals have seen during the past summer their women in mini-skirts exhibiting their legs and now they see the ‘ye-yes’ with manes walking through their streets and avenues … Is it to be deduced that youth, surpassing adults, ha(ve) created a super-European Common Market of tastes and feelings? That area that perhaps begins in Great Britain, passes through Paris, Rome, Zurich, Warsaw and goes around the United States, where a delinquent, brutal, drugged youth proliferates, practicing tribal forms of the community of goods, women, tastes and love of violence. (López Riocerezo, 1970, pp. 139–140)

Los Golfos (Saura, 1959) is a film by Carlos Saura that tells the story of a youth gang on the outskirts of Madrid in the fifties. A young man who aspires to be a bullfighter organises a robbery with his colleagues to raise funds for his debut, with the usual tragic end. The film describes the beginnings of rural-urban migration just before ‘developmentalism’ – the incipient arrival of musical rhythms and international fashions but native slang and forms of organisation – began to predominate. The genre of films about gangs had its heyday in Spain during the democratic transition (1976-1985), following a series of B movies about the phenomenon of the ‘*quinquis*’ – gangs of juvenile pre-delinquents, mostly of gypsy origin, of fast life and tragic end, which was spurred by the heroin market. The significant thing about this genre, exploited by directors such as José Antonio de la Loma in films like '*Perros callejeros*' (De la Loma, 1977) , is that some of the protagonists themselves had participated as actors (such as El Torete and El Vaquilla) who ended up assuming their own tragic roles, becoming antiheroes in reality and fiction.

In 1970, Father López Riocerezo published the book *Problemática mundial del gamberrismo y sus posible soluciones* (The global problem of hooliganism and its possible solutions). This addressed the study of indigenous youth gangs in Spain in the sixties. The author was a religious individual who had obtained some success with publications such as *Génesis del joven rebelde* (Genesis of the young rebel) and *Intenta hacerte hombre* (Try to make you man), continuing the prolific production of ‘edifying literature’ that, during the Franco regime, guided many generations of boys and girls in their intricate ‘opening to life.’ Starting with a curious mixture of the Social Doctrine of the Church, empirical sociology, criminal anthropology, journalistic documentation, and moralising reformism, the author maintains that gangs are nothing more than a new and dangerous type of hooliganism that threatens to undermine the foundations of civilisation:

On another occasion, we wondered, with natural suspicion, whether our Western civilization was threatened by the vertical invasion of a new generation reluctant to (adhere to) any moral code. The acts of juvenile delinquency, which are so profusely recorded in the pages of events, are nothing more than outposts of an anarchic and primitive era, which makes use of numbers, groups and anonymity (…) The underlying evil does not lie in the external characteristics of these boys: their bizarre life, their extravagant hairstyle(s), their taste for bustle, their fondness for rock and roll or twist, their fervor for speeding and their grouping in gangs. The real problem is that they are undisciplined boys, without ideology or morals, friends of debauchery and whose jokes pass (are) on the edge of the asocial, so they easily slide into crime. (López Riocerezo, 1970, p. 17).

Hooligans, *blousons noirs*, teddy boys, *vitelloni*, *raggare*, rockers, beatniks, *macarras*, hippies, *halbstarkes*, provos, *yé-yés*, rock’n’rollers, *pavitos*, etc., are varieties of the same species: deviant youth, the ‘rebels(s) without a cause’. The nearly 300 pages of the pamphlet describe each of these groups throughout the world: teds, mods and rockers are characterised by ‘long hair and disheveled dress in clear opposition to ancestral customs’; *blousons noirs* ‘by their unerring attire of a black jacket or shirt (and who) establish their headquarters in shanties as dirty as they are smelly’; the hippies, ‘a youth animated by a strange mystique and a terrible power of sex and luxury, of drugs and jazz,’ and so on. Of course, thank God, Spain was still sheltered from this threat:

In Spain, for example, we have a relatively lower rate than countries of the same degree of civilization, perhaps due to the historical constant, the weight of the centuries and family tradition, which, as we know, constitute a baggage that one cannot easily get rid of. (*Ibid*., pp. 9-11)

In conclusion, before proposing a series of reformist measures, the author acknowledged that all this was closely related

to the transformation of a society (with a) rural or agrarian culture into (an) industrial and post-industrial (one). When this step is made quickly, a cultural and sociological crisis occurs, as if the channels of integration of the individual in the norms of society are blocked. (*Ibid*., p. 244)

And indeed, the transformation that the country underwent in the sixties was rapid and profound: development plans offered us industrialisation, urbanisation, television, consumer goods, and tourism, all for the same price (paid in large part by the foreign currency associated with tourism and thousands of emigrants to Europe). Likewise, the factors that fostered the growth of youth culture throughout the world also ended up occurring here: secondary education, mass media (radio, television), youth consumer market (music, fashion, entertainment venues), etc. As a result of the emigration processes, in the suburbs of the big cities grew youth gangs that, built their culture with the crumbling fragments of their rural and southern culture of origin and the suburban and proletarian world of the industrialised areas of destination. Many of these gangs became famous, such as the Angelitos Blancos (White Angels) of Cornellà. However, the literature to which they gave rise was only concerned with describing, in a condemnatory tone, their criminal side. On the other hand, with tourism and the thriving leisure market, the new customs of the young people of the West began to spread among the young people of the middle classes. At first, harmless musical fashions and wearing jeans. Later, more dangerous countercultural movements. In coastal areas, especially in Ibiza and Formentera, the hippy movement created one of its main strongholds and began contaminating native youth sectors. However, the differences were obvious: here, the *welfare state* was not known, and the Franco regime left less room for dissent and, at the same time, forced many of these movements to adopt explicitly politicised forms.

In the eighties, after the democratic transition, the hegemonic model of ‘*banda*’ (gang) was popularised under the label of ‘*quinquis*.’ With the economic crisis that affected a significant proportion of youth and the influx of heroin to the urban peripheries of the big cities, the more or less harmless neighbourhood gangs disappeared, and criminal gangs emerged that B movies popularised (De la Loma, 1977). The gangs are not, therefore, an ‘imported phenomenon’ – as some have recently maintained – but are related to internal and older migratory and suburbanisation processes.

## From ‘Urban Tribes’ to ‘Latino Gangs’

We, young Latinos, want and need you to value our cultures and help us integrate into society, having confidence and not labeling the Latino youth as part of a gang or criminal gang. (Queen Melody, paper presented at the seminar ‘Young Latinos: public space and urban culture,’ Barcelona, November 2005)

During the first decade of the twenty-first century, a strong wave of migration from Latin America to Spain took place. At first, it was strongly feminised immigration destined for the service sector and home care. In a second phase, the sons and daughters of these women who had migrated alone also arrived through family reunification. Many were minors who had grown up in their place of origin in the care of relatives or grandmothers. However, young adults also arrived, escaping the economic or political crises of their countries of origin in search of new opportunities. With them came their groups of belonging, which the media baptised with the nickname ‘*bandas latinas*’ (Latino gangs) after some tragic events.

A few months after the death of Ronny Tapias, in 2003, the director of the Prevention Service of the Barcelona City Council commissioned me to carry out a study on young people of Latin American origin in Barcelona and the problem of gangs. There were two police reports – one from the local urban guard and another from Mossos d'Esquadra, the Catalan Police – but they were based on partial statements and information from the internet. The reason for commissioning the study was to go beyond stereotypes and analyze without prejudice the reality of these young people who had arrived in the last five years to Barcelona and its metropolitan area. During 2005, I formed a team that conducted an in-depth field investigation. We started with interviews and focus groups with a hundred adolescents from various high schools in Barcelona, so-called generation 1.5 – that is, minors born in their places of origin, separated at some point in their childhood from their parents, mainly from their mothers, who decided to emigrate to Spain in search of a better future, and subsequently regrouped. We also interviewed professional educators, police, social workers, etc., who gave us their opinions on the gangs. At the beginning of the fieldwork, the gangs appeared as ‘ghosts’ – a series of myths and legends that the young people repeated: almost no one declared themselves a member, although some sympathised.

Near the end of the study, in June 2005, a fortuitous event allowed us to contact them. For a few months, a group of Latinos called STAE Nation had been meeting in a Youth Center in Barcelona. The director of the *Casal* (a youth center) communicated this to the City Council, which, when looking for information, discovered that the meaning of the acronym STAE (*Sagrada Tribu Atahualpa Ecuador*) was linked to the Latin Kings. Although the first temptation was to expel the group from the premises, the Prevention Services director thought it could be an occasion to contact them and called me. After a series of adventures prior to talking to their representatives, I was able to incorporate them into the study and start an intense mediation experiment with the participation of the two supposedly rival groups: the Latin Kings and the Ñetas. In the book that reported on the study (Feixa et al., 2006), I proposed differentiating the following five categories:^5^

1. Bandas (Gangs) (groups, not necessarily juveniles, that are structured around criminal activities, with little symbolic elaboration);
2. Pandillas (youth groups with a local, territorial base, usually structured around leisure and more extraordinarily around illicit activities);
3. Styles (youth groups of a global nature, not structured or cohesive, based on music and aesthetics);
4. Associations (youth groups with a greater degree of complexity and supralocal character);
5. Nations (transnational youth groups structured with different degrees of cohesion and with a strong symbolic and identity component).

As a conclusion to the report and the book, I proposed the following decalogue, which I still consider valid (*Ibid*., 2006):

1. Most Latino youth do not belong to youth organisations.
2. Most youth who belong to Latino youth organisations are not violent.
3. Latino youth organisations are not criminal organisations.
4. Youth who are part of Latino youth organisations may be involved in illicit activities.
5. Youth organisations are no longer exclusively Latino.
6. Youth organisations are no longer exclusively male.
7. Youth organisations do not control territories, but they can join them.
8. Youth organisations can evolve into social and cultural movements.
9. Youth organisations can only evolve from within.
10. Some youth organisations want to and can evolve.

On 14 September 2006, a new youth entity was entered into the register of associations of the Catalan government. The Cultural Organisation of the Latin Kings and Queens of Catalonia was presented at a Youth Centre in Barcelona. The event would not have aroused much interest if not for the fact that such a name evoked a social imaginary until a few months previously that was synonymous with something almost diabolical: the dangerous gang of the Latin Kings. The meeting was attended by almost a hundred journalists of all types of media (including almost all televisions and the correspondents of most American newspapers), who witnessed, astonished, the coming out of the closet of a handful of Kings and Queens, with its president, Queen Melody, at the front. At the end of the event, those responsible for the Youth Council of Barcelona commented ironically that the activities of the rest of the city’s youth associations had never aroused so much interest. What had happened so that a dangerous gang of criminals could have become a harmless cultural association? Were they the same as those in the United States who were part of the largest and most feared gang, and those in Madrid a judge proposed to declare an ‘illicit association’? What was behind this scenery of yellow and black necklaces and crowns?

Following the ‘ghost’ of the gangs, an ignored presence, thousands of boys and girls arrived in Barcelona from the late nineties onwards (thanks, fundamentally, to various processes of family reunification), (banished) from their places and social networks of origin at the most critical moments of their lives (the always difficult transition to adult life), and were confronted in their place of destination with terrified adults (over-occupied mothers, often absent fathers, insecure teachers, and social workers, neighbours in fear) and a state of legal and institutional liminality. Behind this disturbing presence is a specter: that of new forms of sociability that cross geographical and temporal borders to reconstruct global identities that we continue to confuse with traditional gangs. According to official data from the Barcelona police, the number of young people who belonged to ‘Latino gangs’ in that period ranged between 400 and 1,000 (primarily men). Without delving into the fact that their organisations are also made up of non-Latin-American members, the former would only represent between 1% and 2% of the youth population of these nationalities (between 2% and 4% if we discard those under fifteen). Therefore, it would be pertinent to ask ourselves the following and reflect on emerging problems around this issue: Why does the dominant imaginary build the stereotype of the young Latin American on the scarce 2% that supposedly belong to a gang, making invisible the remaining 98%? That is, why encourage the spread of the phenomenon among young people not previously attracted to this way of life, which becomes a refuge for a questioned identity – a self-fulfilling prophecy?

Within the framework of this participatory action research, a complex but extraordinarily interesting process was initiated. Previously, rival groups – the Latin Kings and the Ñetas – began to implement their project of being recognised as associations. With advice from the Institute of Human Rights, they drafted statutes faithful to their principles and Catalan laws. For several months, they discussed the draft in grassroots meetings (called ‘chapters’) and assemblies (called ‘universals’). In the case of the Latin Kings & Queens, the debate coincided with the debate about the Catalan statute of autonomy, which led to funny situations – for example, concerning the name of the association: at first, the members of the group did not understand why they could not use the official name (Almighty Latin Kings and Queens Nation). While legal advisors assured them that the name was not the thing, the definitive argument was knowing that Catalonia had not been recognised as a nation in the Statute approved in the parliament in Madrid. One Sunday in May 2006, a priest who had long been committed to the migrant community and who had welcomed Kings and Queens in his parish sent me an SMS after attending the final vote that said: ‘I do not know if we will have a Statute, but we already have statutes!’ Soon after, the statutes were submitted to the registry of associations under the name the Cultural Organisation of Latin Kings and Queens of Catalonia, were finally recognised by the Department of Justice in July 2006, and soon appeared in the press.

What did this recognition entail? Can a Latino gang become a cultural organisation? According to the legal advisers who have been involved in the issue, rather than ‘legalization,’ we should speak of ‘constitution of association’ (since these groups were not previously illegal, but instead ‘alegal,’ like most informal youth groups). However, the social dynamic that the process has ushered in is more important than this legal recognition: identities hitherto proscribed are now accepted; the stigma becomes an emblem. The boys and girls who until then were underground can now be out in the open (not unlike processes previously experienced by prohibited political groups under Franco’s dictatorship). The simple process has already had positive effects: acts of violence have declined, and members have turned to cultural creativity: sports championships, theatrical performances, the making of a documentary film, and even the recording of a hip-hop and reggaeton CD. The most successful intercultural mediation project, in which Latin Kings, Ñetas, and other young people from Barcelona participated, was promoted by a youth centre in Nou Barris, a neighbourhood of old and new migration, and supported by an alternative record label. This resulted in the production of a CD, a documentary film, and a book published under the title *United for the Flow* (VVAA, 2008). Similar processes occurred in Alicante, the Balearic Islands, and Navarre during the same period. On the other hand, in other places such as Madrid and Murcia, where the same groups existed, the approach was to opt for the ‘iron fist’ and a fundamentally police and judicial solution.^6^

After 2010, the situation changed: with the arrival of the crisis, many young Latinos lost their jobs, some returned to their country of origin, and others found themselves in an irregular situation or went through jail. The changes in the Catalan government's Interior Ministry implied a return to the ‘iron fist’ and the end of such attempts at mediation. However, the groups in Catalonia were not outlawed and continued to function. The two that had participated in the process – the Latin Kings and the Ñetas – were pacified, but other smaller groups that had not participated in the previous process emerged. The two most important of these of Dominican origin – the Trinitarios and Dominicans Don't Play – began a confrontation that still exists that caused the media to start talking about the ‘rebound of Latino gangs.’ The reform of the Spanish penal code in 2010, with the introduction of the notions of ‘criminal group’ and ‘criminal organization,’ which were added to the pre-existing one of ‘illicit association,’ ended up consolidating this evolution (Ballesté & Feixa, 2022).

## Discussion: ‘Latino gangs’ as a symptom of moral juvenicide

In our contribution to the book *Juvenicidio* (Valenzuela, 2015), we proposed the notion of ‘moral juvenicide’ in an attempt to conceptualise the process of systematic exclusion of Spanish youth during the post-2008 crisis (Feixa et al., 2015). Pursuing the investigation of different types of violence, we observed that the labour, educational, political, residential, and affective precariousness that affect many young people today could be classified as a type of symbolic violence (Bourgois, 2001). This means

the symbolic disappearance of youth as a social actor, (their) invisibility as (…) protagonist(s) of the public scene and the metamorphosis of the juvenile period, which goes from being a transitional phase to being an intransitive phase, as a result of the failed, pendular or endless trajectories towards adult life. (Feixa et al., 2015, p. 236)

By moral juvenicide, we understand not the physical death of young people but the reduction of their life expectations, future projects, and free development as people; that is, what some authors call ‘drip juvenicide’ (Muñoz, 2015). From this perspective, juvenicide ceases to be something exclusive to peripheral countries with authoritarian political regimes that do not respect human rights or physically mistreat young people but can also be applied to the situation of some central countries where there has been or is taking place a process of the ‘programmed social exclusion of youth’ (Claret, 2013) or adult-centrism (Duarte, 2015). In Spain, these policies of social exclusion may be shown through two ‘parallel’ scenarios: on the one hand, the destruction and precariousness of youth employment (that can be called ‘economic juvenicide’); on the other, the media and cultural stigmatisation of young people (that can be called ‘symbolic juvenicide’). When the two processes converge, we can speak of ‘moral juvenicide’ (Muñoz & Feixa, 2022; Strecker et al., 2018).

We may wonder whether the notion of moral juvenicide is applicable to the ‘Latino gangs’ I have just outlined. From the outset, it should be borne in mind that with youth gangs, there occur three different types of juvenicidal practices: first, what can be called ‘juvenicide from above,’ that is, the process of physical, legal, and moral abuse that the State, security forces, adult institutions or racist groups exert on subaltern youth of migrant origin; second, what can be called ‘juvenicide from below,’ that is, the physical or moral violence practiced in conflicts between rival groups or gangs of a similar origin or social stratum; third, what can be called ‘juvenicide from within,’ that is, processes that are exercised within the group in the form of physical punishment, bullying, gender violence, hazing or the imposition of a certain ‘law of silence.’ While the media tend to pay attention almost exclusively to the last two types of juvenicide – those that are exercised between gangs or within gangs – they almost always dismiss or justify ‘juvenicide from above’ despite this being the only phenomenon that, in a strict sense, deserves such a qualification since it is based on a power relationship that is exercised by the state or hegemonic groups.

In the case of young people of migrant origin, particularly those of Latin American origin, they are propitiatory victims of all the dimensions of moral juvenicide: they have been those most harmed both by economic juvenicide – being cannon fodder of the underground economy, precarious work, low wages, and sometimes labour exploitation – and symbolic juvenicide – victims of racist stereotypes rooted among the population, including sectors of the police, the judiciary, and politics. Other aggravating dimensions could be added here, such as ‘legal juvenicide’ – caused by an increasingly restrictive Aliens Law, which generates situations of ‘alegality’ for many people, including undocumented minors – or ‘educational juvenicide’ – caused by school failure for a very large proportion of the youth of Latin American origin, caused by complicated migratory processes during childhood or adolescence. This involves difficult adaptation to the Spanish school environment, school segregation according to neighbourhood and social class, early school leaving, and even the hidden racism of a sector of the teaching staff. Where there are no means of compensating for such discrimination, this may result in forms of belittling, self-harm, depression, or fatalism compatible with moral juvenicide. In our research with young people of Latin American origin in recent years, we have seen many examples of this, and both the victims and the perpetrators of recent murders may be responding to this pattern. Not to mention cases of structural and institutional racism, or the presumption of guilt that we have witnessed in sectors of the police, the judicial system, the media, neighbourhood entities, and extreme right-wing political groups.

From this perspective, membership of the ‘Latino gangs’ should not be seen as the cause of violence between equals – as falsely presented by the media and certain extremist groups – but may even be interpreted as a ‘magical’ or ‘symbolic’ form of protection and empowerment – in fact, as one of the few resources that these vulnerable young people have to survive or resist, even if this does not solve their structural problems and is sometimes at the cost of harming their peers or themselves.

## Conclusion: from ‘Latino Gangs’ to ‘Youth Gangs’

While there have always (been) gangs, today’s urbanizing world is producing them faster than ever and in myriad forms and shapes. High levels of violence by ‘non-state actors’ such as gangs or terrorists have been disturbing aspects of globalization. The evidence I present in this book leads to the uncomfortable conclusion that gangs are not going away, no matter what we do. (Hagedorn, 2008, p. xxiii)

In 2017, the European Research Council awarded me an Advanced Grant to study transnational gangs as agents of mediation. Since 2018, a team composed of twenty researchers has carried out fieldwork in twelve cities in southern Europe (Barcelona, Madrid, Marseille, and Milan), North Africa (Rabat, Tunis, Algiers, and Djendel), and the Americas (Medellín, San Salvador, Santiago de Cuba and Chicago). In all of them, we have investigated different types of transnational gangs (from criminal organisations to subcultural groups through hybrid models), focusing on good practices of mediation (Feixa et al., 2019, 2022, 2023; Feixa & Sánchez-García, 2022, 2023).

The events reported in Barcelona and Madrid, as well as the most serious events in El Salvador (breaking of the truce between the government and gangs, declaration of a state of alarm, mass arrests, and imprisonment) and Ecuador (massacres in prisons, war between criminal organisations, the assassination of the Ecuadorian leader of the Latin Kings and a presidential candidate), show that, unfortunately, this is a recurring theme. In this article, I have started from this observation, placing it in its historical context, with the intention of providing keys for reading that can increase understanding of the situation and help overcome stereotypes about street youth groups at a time when there is a severe risk of stigmatising all young members of these groups, especially those of migrant background. As can be seen from the debate that took place in the Assembly of the Community of Madrid a few days after the two murders mentioned at the beginning of the paper, there is a risk that the stigma of ‘Latino gangs’ will be replaced by the stigma of ‘youth gangs,’ with the valid argument that many of the members of these groups are no longer born in Latin America and/or have Spanish nationality. However, there is the danger that the stigma of gang membership will spread to broad layers of subaltern youth.^7^

Gangs are not always the problem; they can also be part of the solution. The issue cannot exclusively be addressed with a police or criminal justice approach but requires strong commitment and social investment. Public policies and programmes implemented to address the problem should be guided by research rather than stereotypes, combining *a posteriori* punitive and restorative measures with *a priori* preventive and inclusive measures and *ad hoc* rehabilitation and reintegration measures.

## Notes

1 The Latin Kings & Queens were born on the streets of Chicago in the 1960s. Their official name is the *Almighty Latin Kings and Queens Nation* (ALKQN). In the 1970s, they expanded to New York and other US cities. In the 1990s, they arrived in Ecuador and other Latin American countries due to deportation policies. In 2000, they were reborn in Madrid and later in Barcelona and other cities in Spain and Italy through migration. See Brotherton and Barrios (2003).

2 The Ñetas were founded in a prison in Puerto Rico in the 1970s by a pro-independence leader. The group’s official name is *Asociación Ñeta pro Derechos del Confinado* (Ñeta Association for the Rights of Prisoners). Soon after, they arrived in New York and other American cities. In the 1990s, they arrived in Ecuador, and at the beginning of the new century, they were reborn in Spain and Italy. See Lamotte (2022).

3 The Dominican Don’t Play (DDP) were born in New York in the 1990s and soon expanded to the poor neighborhoods of the Dominican Republic. It arrived in Spain in 2004. See Núñez (2020).

4 The Trinitarios were born in a prison in New York in 1989, then expanded within the US and the Dominican Republic, where they became rivals of DDP. The name refers to the founder of the country in the XIX century, a masonic liberal, which has created some confusion. They arrived in Spain circa 2004. See Núñez (2022).

5 Subsequent events, including the initial raid that led to contact, the process of the constitutionalization of the Cultural Organization of Latin Kings and Queens of Catalonia (2006), and the Musical, Sports and Cultural Association Ñetas (2007), are related in the book *El Rey. Diario de un Latin King* (Feixa & Andrade, 2020, English translation forthcoming).

6 After the success of the Barcelona experiment, a similar attempt was initiated in Genova and Milano (Queirolo Palmas, 2016). In 2007, Ecuadorian President Rafael Correa recognized the *Corporación de Reyes y Reinas Latinos de Ecuador*, with positive effects in the form of a reduction in the homicide rate in the country (see Brotherton & Gude, 2018).

7 In June 2022, the Assembly of Madrid set up a Commission of Inquiry into Youth Gangs. As the Principal Invetigator of TRANSGANG I was summoned to intervene in the constitutive session at the proposal of four groups of different political persuasions.
